# Atypical Stevens-Johnson Syndrome Associated With Mycoplasma Pneumoniae

**DOI:** 10.7759/cureus.21825

**Published:** 2022-02-02

**Authors:** Ramin Beheshti, Bryan Cusack

**Affiliations:** 1 Pediatrics, Penn State Health Milton S. Hershey Medical Center, Hershey, USA

**Keywords:** delayed diagnosis, diagnostic medicine, atypical rash, mycoplasma pneumonia, steven johnson syndrome

## Abstract

*Mycoplasma pneumoniae* primarily causes atypical pneumonia in children and young adults. 7%-8% of patients with *M. pneumoniae* infections may experience extra-pulmonary manifestations, including *M. pneumoniae*-associated Stevens-Johnson Syndrome (SJS), also known as atypical SJS. In recent literature, there have been a few reports of isolated mucositis in children with *M. pneumoniae* infections. Due to significant overlap with several diseases, including autoimmune disease and infections, atypical mucositis associated with *M. pneumoniae* is often a diagnostic challenge. In addition, due to limited cases of *M. pneumoniae*-associated SJS, there is no established standardized treatment guideline that has been shown to reduce hospitalization duration and/or disease progression associated with *M. pneumoniae*-associated SJS. We report a case of isolated mucositis in the absence of cutaneous involvement in a 10-year-old patient with an acute *M. pneumoniae* infection. Examination revealed erythematous ulcerations of his lips and pharynx with patchy exudates and bilateral submandibular lymphadenopathy. Laboratory investigation revealed a negative respiratory polymerase chain reaction (PCR) panel, which included *M. pneumoniae*. Further testing revealed a positive *M. pneumoniae* immunoglobulin M (IgM) titer on enzyme immunoassay. The diagnosis of atypical SJS was made secondary to *M. pneumoniae*. Treatment was initiated with systemic steroids and oral antibiotics. Limitations in diagnostic testing for *M. pneumoniae* in combination with non-specific clinical presentation make for challenges in confirming this pattern of SJS due to a primary *M. pneumoniae* infection. In this case, serological testing confirmed our suspected diagnosis, which guided treatment and helped reveal some of the difficulties in diagnosing and managing *M. pneumoniae*-associated SJS.

## Introduction

*Mycoplasma pneumoniae* is a common infectious cause of community-acquired pneumonia; however, isolated mucositis has been reported in children with *M. pneumoniae* infections [1]. SJS is a type four hypersensitivity reaction to cutaneous and mucosal tissue involving the stimulation of cytotoxic CD8+ T cells and helper CD4+ T cells [2,3]. Drug reactions account for 50% of typical cases, which usually present with targeted skin rash in addition to mucositis [3]. Recently several reports of isolated mucositis (atypical SJS) have been reported with infections [4]. One such causative agent of atypical SJS is *M. pneumoniae* [5]. *M. pneumoniae* is an obligate intercellular organism associated with pneumonia in pediatric and young adult populations [1]. In addition to respiratory involvement 7%-8% of cases result in extrapulmonary manifestation, including arthritis (<3%), hepatitis (<5%), pericarditis (<1%), hemolytic anemia (<5%), aseptic meningitis (<1%), and SJS (<1%) [6]. *M. pneumoniae*-associated SJS (atypical SJS) is clinically diagnosed based on the distribution of the rash, along with associated clinical findings [4]. Typical SJS typically begins with a fever and progresses with non-specific symptoms that may overlap with various infectious or inflammatory processes [4]. As it progresses, patients may experience mucosal desquamation around the mouth and anogenital regions [7]. In typical SJS, cutaneous involvement usually includes the face, arms, legs, and back and appears in the form of targetoid lesions [4]. Typical SJS is a clinical diagnosis; however, in atypical SJS sources of infectious etiology may be elicited, with further laboratory workup to mitigate timely treatment. Serology is the gold standard test for laboratory diagnosis [8]. Polymerase chain reaction (PCR) is a rapid and helpful test as well, especially when combined with serology [8]. Clinicians often rely on PCR testing to exclude *M. pneumoniae* due to the rapid availability of results; however, this test has a sensitivity of 60%-70%, which may result in false negatives [8]. Serological testing has a sensitivity of 90%. Unfortunately, serological testing is often excluded in such presentations as the non-specific clinical presentations may often mislead clinicians to work up a wide spectrum of diseases that affect the mucous membranes. In addition, *M. pneumoniae*-associated mucositis is extremely rare, so limitations in awareness of *M. pneumoniae*-associated mucositis may lead to delayed testing [8]. Therefore, appropriate management and treatment may be delayed due to diagnostic challenges. Therefore, early diagnosis is important in guiding the management and treatment of the underlying infectious cause for SJS. In addition to diagnostic challenges, there is currently no established standardized guideline to help clinicians manage *M. pneumoniae*-associated SJS [8]. Antibiotics may help treat *M. pneumoniae* infections, but no clear relationship between antibiotics and progression of mucositis has been established [9]. The use of steroids in *M. pneumoniae*-associated SJS is controversial, with no evidence of reduced mortality or hospital duration based on current literature [8]. Supportive care, including nutrition optimization and pain management, has been associated with improved patient satisfaction but has not been associated with reduced hospitalization duration [9]. We report a case of isolated mucositis in the absence of cutaneous involvement in a pediatric patient with *M. pneumoniae*. We demonstrate difficulties in making this diagnosis due to a non-specific clinical presentation and the insensitivity of the most utilized methods of detecting *M. pneumoniae*. We also highlight the absence of a standardized guideline for the management of patients with *M. pneumoniae*-associated SJS and discuss the outcome of our patient's specific treatment regimen. 

## Case presentation

**Figure 1:**
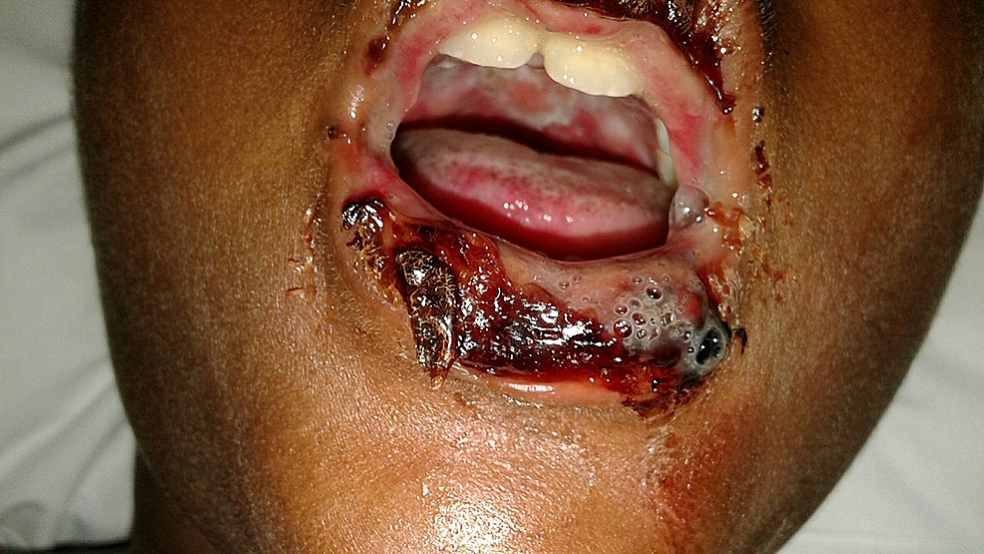
Initial examination revealing erythematous ulcerations of the lips and pharynx with patchy exudates

A 10-year-old male with no significant past medical history initially presented with 1-2 days of bilateral conjunctivitis and mucositis with intermittent fevers for two weeks. Fevers had not occurred daily but had not resided despite daily antipyretics. Following the initial onset of fevers, he developed a dry cough, congestion, and rhinorrhea. He had not experienced increased work of breathing or wheezing. His parents reported no history of asthma. He developed mouth sores with severe odynophagia 1-2 days before admission. He had no skin rash or extremity/facial swelling. He was not taking any scheduled prescription medications at home. On physical examination, his vitals are as follows: temperature, 97.8F (36.5C); blood pressure, 84/55 mm Hg; heart rate, 122 beats/min; respiratory rate, 27 breaths/min. Examination revealed erythematous ulcerations of his lips and pharynx with patchy exudates and bilateral submandibular lymphadenopathy (Figure 1). In addition, he demonstrated bilaterally injected conjunctiva with purulent drainage. No skin lesions or rashes were present. The rest of his examination findings are unremarkable. Laboratory investigation revealed a negative respiratory PCR panel that tested for common viruses and bacteria, including *M. pneumoniae*. Additionally, laboratory investigation revealed elevated C-Reactive protein at 3.1 mg/dL (normal range: <10mg/dL) and erythrocyte sedimentation rate at 58mm/hr (normal range: 3-13mm/hr) and an unremarkable complete blood count (CBC). Further testing revealed a positive *M. pneumoniae* IgM titer on enzyme immunoassay, which resulted on day three of his hospitalization. The diagnosis of atypical Stevens-Johnson Syndrome (SJS) was made secondary to *M. pneumoniae*. The patient was started on a seven-day course of IV Methylprednisolone at 1mg/kg/day divided by four daily doses and a five-day course of Azithromycin at 500 mg orally on day one, followed by 250 mg on days 2-5. His symptoms improved quickly and resolved three days after the onset of treatment. He was discharged home upon completion of IV steroids with complete resolution of his mucositis.

## Discussion

SJS is often characterized by skin, mucosal, and genitourinary lesions [2]. This case presents a unique atypical presentation of SJS characterized by isolated oral mucositis in the absence of cutaneous lesions. This atypical presentation of SJS has been linked to *M. pneumoniae*, which has been reported as an extra-pulmonary manifestation in children and young adults aged 6-19 [1]. Extrapulmonary manifestations of *M. pneumoniae* are rare but have been reported in previous literature [1]. *M. pneumoniae*-associated SJS without cutaneous involvement is extremely rare and poorly understood. Establishing the diagnosis of atypical SJS is challenging due to clinical similarities with more common diseases, including autoimmune diseases and infections. Additional infectious agents, including HSV, group A streptococcus, EBV, and Hepatitis B virus, have been associated with isolated mucositis [1]. Limitations in diagnostic testing for *M. pneumoniae* in combination with non-specific clinical presentation make for challenges in confirming this pattern of SJS due to a primary *M. pneumoniae* infection [8]. Given the association between SJS and *M. pneumoniae*, serological diagnostic testing for *M. pneumoniae* should be considered in pediatric patients with isolated mucositis, particularly in cases where the clinical diagnosis of SJS may be more uncertain. Two factors should be evaluated when choosing the best diagnostic test, accuracy and time to obtain results. *M. pneumoniae* is a small ubiquitous microorganism and lacks a cell wall, making it difficult to detect by gram staining [1]. Cultures can confirm their presence but take 2-6 weeks, making them ineffective in guiding treatment during an acute presentation [10]. PCR is a quick diagnostic method but has a sensitivity of 60%-70%, leading to a high rate of false negatives as with our patient. Serology testing specifically complements fixation assays, and indirect immunofluorescence has better sensitivity and specificity, 80%-90% and 92%-100%, respectively [8]. However, this method takes several days and can increase the cost of hospitalization [8]. There is currently no established standardized guideline to help clinicians manage *M. pneumoniae*-associated SJS [8]. Often antibiotics are initiated to treat *M. pneumoniae*, but the dosing and duration are not well established. The use of steroids in *M. pneumoniae*-associated SJS is controversial, with no known studies that have demonstrated reduced mortality or hospital duration with the initiation of steroids. Supportive care, including nutrition optimization and pain management, has been associated with improved patient satisfaction but has not been associated with reduced hospitalization duration [8]. Therefore, treatment of *M. pneumoniae*-associated SJS remains poorly understood, with no objective data to provide evidence-based guidelines for clinicians to manage such patients. In this case, serological testing confirmed our suspected diagnosis and guided effective treatment. We highlight that the symptoms resolved three days after the initiation of a seven-day course of systemic steroids (Methylprednisolone) and a five-day course of oral antibiotics (azithromycin). We suggest that when initiated early in disease progression, the combination of steroids and antibiotics may reduce the duration of hospitalization and improve clinical outcomes for patients with *M. pneumoniae*-associated mucositis, as in our case. 

## Conclusions

Atypical SJS differs from classical SJS with regards to clinical presentation and etiology. *M. pneumoniae*-associated SJS has been reported in the literature but is extremely rare and therefore poorly understood. *M. pneumoniae*-associated SJS can mimic various autoimmune and infectious conditions, which makes this a diagnostic challenge. Currently, no established treatment regimen has been shown to reduce hospitalization duration and/or mortality associated with *M. pneumoniae*-associated SJS. This case outlines the diagnostic challenges in identifying *M. pneumoniae* as the causative agent of atypical SJS and demonstrates shortened hospital duration and reduced disease progression following the initiation of methylprednisolone and azithromycin. Cases that demonstrate reduced hospitalization duration and disease progression with timely diagnosis and effective treatment may improve objective data and help establish a standardized, evidence-based treatment guideline with guidance on how to care for patients with *M. pneumoniae*-associated SJS.
